# Reconstruction of patients' treatment preferences in disorders of consciousness: a systematic review

**DOI:** 10.1186/s12910-025-01241-1

**Published:** 2025-07-04

**Authors:** Niek Kok, Willemijn van Erp, Marjan J. Meinders, Jelle van Gurp

**Affiliations:** 1 Department of IQ Health, Radboud University Medical Center, Nijmegen, the Netherlands; 2 Department of Primary and Community Care, Radboud University Medical Center, Nijmegen, the Netherlands; 3 Accolade Zorg, Bosch en Duin, the Netherlands; 4 Libra Revalidatie & Audiologie, Tilburg, the Netherlands; 5 Department of Neurology, Radboud University Medical Center, Nijmegen, the Netherlands

**Keywords:** Disorders of Concsiousness, Ethics, Treatment preferences, Epistemology, Systematic review, Clinical decision-making

## Abstract

**Background:**

Patients with disorders of consciousness (DoC) are unable to partake in the decision making process concerning their treatment. In the process of medical decision-making, which in DoC often concerns life-and-death decisions, surrogates and healthcare professionals may try to reconstruct the treatment preferences of these patients. We aimed to identify which values and criteria have been used in various national care contexts to reconstruct the treatment preferences of incapacitated DoC patients and how reconstruction is conducted in practice.

**Methods:**

This is a systematic review of the of conceptual and empirical ethical literature. A search was performed in seven databases (Pubmed, Web of Science, Embase, the Cochrane Library, CINAHL, PsychINFO, and Sociological Abstracts). We used thematic analysis to identify values and criteria for reconstruction of treatment preferences.

**Results:**

8.591 records were screened. In total, 17 conceptual studies and 13 empirical studies on preference reconstruction were included. We identified three normative-epistemic viewpoints on reconstruction of treatment preferences: a correspondence view which aims to respect personal autonomy and prioritizes the accuracy of reconstructed preferences; a coherence view which aims to respect personal identity and prioritizes the consistency of the preferences with the patient’s lifeworld; and a communitarian view which aims to respect community and prioritizes the ongoing relation of a patient with family and friends. These views diverge on the problem of what makes for a good process of preference reconstruction. Additionally, treatment preferences of patients in DoC are inferred based on either past oral statements or on observations of patients’ current behavior. The criteria that guide reconstructive efforts may evolve, especially when patients improve from UWS to MCS and when reconstructed preferences based on past statements and patient’s current inferred psychological mental states steer treatment in mutually exclusive directions. There is no current standard approach to reconstructing treatment preferences in incapacitated DoC patients.

**Conclusions:**

We recommend physicians to ask diversely formulated questions that stimulate surrogates towards giving multiple and rich answers. Simultaneously, physicians are advised not to overly test a surrogate’s testimony because this may lead to an erosion of trust.

**Supplementary Information:**

The online version contains supplementary material available at 10.1186/s12910-025-01241-1.

## Background

Brain injured patients with disorders of consciousness (DoC) – such as coma, unresponsive wakefulness syndrome (UWS) or the minimally conscious state (MCS) – are by definition unable to partake in the decision making process concerning their own treatment (Table 1) [1, 2]. They may remain unable to communicate for months to years [3–5]. Return of decisional capacity along the course of recovery is uncertain, but these patients are likely permanently neurologically compromised so that that they will never fully recover to their pre-injury baseline. Meanwhile, they require life-sustaining treatments (LST) that are often intensive and invasive, e.g. mechanically assisted ventilation, cannulation, artificial nutrition and hydration (ANH), or a cerebral shunt. Withholding and withdrawing treatment may undermine the patient’s potential to recover from DoC or result in the patient’s death. However, continuation of treatment may also lead to an unwanted outcome, for example a chronic DoC or a conscious state with severe neuropsychiatric symptoms.

**Table 1:** Disorders of consciousness

General level of consciousness	Characteristics
Coma	1. Patient has no sleep–wake cycle, eyes are permanently closed 2. Poor or no control of fundamental bodily functions such as breathing, body temperature, blood pressure 3. No arousal and no awareness 4. Usually no reaction to stimuli
Unresponsive Wakefulness Syndrome	1. Patient has sleep–wake cycle 2. Vital functions are maintained independently 3. No behavioural evidence of awareness 1. Only reflex responses
Minimally Conscious State—Minus	1. Patient has sleep–wake cycle 2. Vital functions are maintained independently 3. Discernible behavioral signs of awareness, such as visual pursuit and affective reactions 4. Low-level intentional behavior such as: a. No sign of language function b. No functional communication or functional object use
Minimally Conscious State—Plus	1. Patient has sleep–wake cycle 2. Vital functions are maintained independently 3. Discernible behavioral signs of awareness, such as visual pursuit and affective reactions 4. High-level intentional behavior such as: a. Evidence of language function, e.g. inconsistent command following b. Some intentional communication, intelligible verbalization, purposeful reaching for objects

Most patients with DoC have no written advance directives (AD) [6–8]. Additionally, these patients are often young and have not engaged in pre-hospitalization conversations aimed at identifying their treatment preferences or otherwise advance care planning. Therefore, surrogates have to make decisions on their behalf – often based on their own impressions of the patient’s treatment preferences [2, 9, 10]. Compared to other situations in which surrogate decisions are necessary, surrogate decision making for patients with DoC is unique because it is likely that these patients will be permanently incapacitated and their mentation is unlikely to improve. In most countries’ legal systems, surrogates of patients with DoC either have to apply the substituted judgment standard or the best interest standard (Table 2) [2, 10–13]. Within both decision making frameworks, surrogates have to reconstruct the patients’ treatment preferences in order to ensure patient-centered care [12, 13]. Whereas there are standards to elicit patient values and treatment preferences as part of advance care planning [14–16], there are none that guide physicians towards a high quality reconstruction of a patient’s treatment preferences when no careful process of advance care planning took place. Little is known about actual moral routines of reconstruction of treatment preferences in clinical practice. From a reconstruction standpoint, patients with DoC are a difficult category, because brain injury often results from sudden, unanticipated accidents. This means that persons who develop DoC have almost never given explicit forethought about treatment preferences. It is also not known which information surrogates actually use to reconstruct treatment preferences, nor how healthcare professionals help support reconstructive efforts during decision making for patients with DoC. To address these gaps, this systematic review aimed at identifying:

1. Which values and criteria have been used internationally to reconstruct the treatment preferences of incapacitated DoC patients;
2. How reconstruction for DoC patients is conducted in actual practice.

**Table 2:** Decision-making standards for patients with DoC

The substituted judgement standard asks of surrogates to decide how the patient would have decided herself, guided by a question such as:
‘would this patient have wanted this treatment’?
The best interest standard requires surrogates to weigh the patient’s reconstructed treatment preferences against their overall wellbeing. The guiding question in the best interest standard would be:
‘is this treatment the best option for this patient’? [2, 10, 11].

## Methods

### Design

This is a systematic review of empirical ethical literature [17, 18]. A search was performed in seven databases (Pubmed, Web of Science, Embase, the Cochrane Library, CINAHL, PsychINFO, and Sociological Abstracts) on November 6th, 2023. We followed the Preferred Reporting Items for Systematic Reviews and Meta-Analyses (PRISMA) guideline. The search strategy was designed with the help of a librarian and included terms related to DoC based on an earlier study [19] combined with search terms for shared decision-making, surrogate or proxy decision-making or substituted judgement (Supplement 1).

### Study selection

All retrieved records were imported into EndNote. Duplicates were removed. NK and MJM assessed the predefined eligibility criteria (Supplement 2) and evaluated a random sample of 100 titles and abstracts of the retrieved records to refine the inclusion and exclusion criteria. Thereafter, NK and MJM independently screened titles and abstracts of all records to determine whether a study met the inclusion criteria. Importantly, studies were only included if it concerned patients with DoC, which includes coma, the unresponsive wakefulness syndrome (UWS), and the minimally conscious state (MCS) [19].

### Quality assessment

All texts were screened by the first author (NK). Records meeting the eligibility criteria were categorized into normative contributions and empirical contributions. NK and one of three reviewers (WE, MJM, JG) independently assessed the quality of empirical contributions using the mixed-method appraisal tool for qualitative, quantitative, or mixed-methods studies [20], and the Critical Appraisal Checklist for Case Reports [21]. Each record was discussed until consensus was reached about both its inclusion and its appraisal. Supplement 3 provides an overview of the quality of the studies.

### Analysis

Both conceptual and empirical studies may propose or discuss values and criteria to evaluate or guide reconstruction of treatment preferences, a thematic analysis was conducted on all studies using Atlas.ti [17, 18]. In each article any value, norm or criterium related to reconstruction of treatment preferences was coded. Thereafter, codes were categorized into larger themes.

## Results

**Fig. 1:**
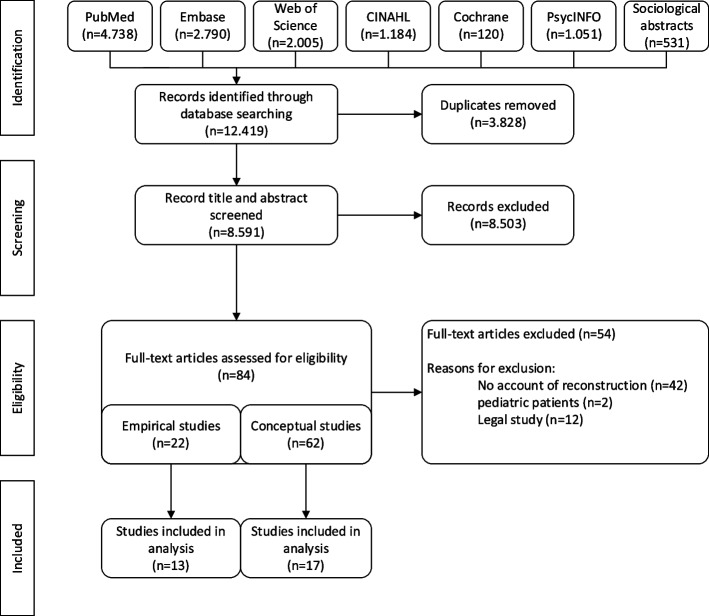
PRISMA flow diagram

Initially, 12.419 records were retrieved. After duplicate removal, 8.591 records remained for screening of title and abstract, after which 84 studies remained (Fig. 1). After critical appraisal, thirty articles were included in this review. We describe the study characteristics and present the conceptual and empirical materials separately.

### Characteristics of selected studies

The included studies, published between 1985 and 2023 [7, 8, 12, 13, 22–47], were conducted in the United States of America (*n* = 14), the United Kingdom (*n* = 5), Germany (*n* = 3), the Netherlands (*n* = 2), Canada (*n* = 1), China (*n* = 1), Finland (*n* = 1), Italy (*n* = 1), Japan (*n* = 1), and Norway (*n* = 1). We found 17 conceptual studies and 13 empirical studies. Most conceptual studies were from the USA and the United Kingdom, meaning that the results of this literature review mostly reflect a Western and specifically Anglo-Saxon perspective on reconstruction of treatment preferences.

### Conceptual studies on reconstruction of treatment preferences

Table 3 reports the values and criteria used to guide reconstruction of treatment preferences in DoC. Reconstruction of treatment preferences was mainly cast as an epistemic normative problem [12, 23, 30, 36]. We distinguish a correspondence and coherence approach to how reconstruction of treatment preferences takes place.

**Table 3:** Values applicable to the input used for reconstruction, or the product of reconstruction

**View**	**Values**	**Relevance to the input used for or to reconstructed treatment preferences**	**Reference**
Correspondence	Accuracy^1^ or preciseness	Not further defined	[13, 22, 36, 42, 47]
	Applicability or relevance	Not further defined, but is always mentioned in relation to the patient’s current circumstances	[8, 13, 28, 42, 43]
	Clarity	Not further defined	[8, 25–27, 33, 39, 42, 46]
	Convincingness	Not further defined, but a written advance directive would be convincing	[26, 27, 33, 46]
	Correctness	Not further defined	[8, 29]
	Verifiability, corroborativeness	It must be possible to check evidence of past statements made by the patient against other evidence	[27, 33, 46]
	Credibility	Not further defined	[30, 46]
	Demonstrability	Not further defined, but a written advance directive would be demonstrable evidence	[26, 27]
	Explicitness	Past statements must explicitly indicate what must be chosen	[8, 26, 28, 29, 42, 46]
	Factuality, validity	Whether reconstructed treatment preferences have a basis in fact	[8, 12, 26, 29, 43, 45]
	Frequency, repetitive statements	Whether a patient has repeatedly or recurringly expressed preferences	[39, 44]
	Genuineness, sincerity	Past statements must reflect what the patient actually wanted	[13, 22, 26]
	Informedness	In making past statements, the patients should have been informed of PDoC	[13, 33–35]
	Non-casualness	Statements used for reconstruction should not be casually made	[26, 46]
	Multiplicity of sources	It is important to assess multiple sources of evidence or input to check the validity of reconstructed treatment preferences	[27, 36, 45]
	Reliability	Used mainly in the context of patients with MCS, to assess whether a patient consistently expresses the same thing; whether the patient has repeatedly made the same statement or behaved in a certain way	[34]
	Specificity	Not further defined, but is always mentioned in relation to the patient’s current circumstances	[13, 32, 33, 39]
	Stability	Past statements should still be valid at some future time	[13, 33]
	Tangibility	Evidence of a patient’s treatment preferences must not be putative	[22]
	Truth-value	Whether past statements that indicate treatment preferences can be determined to be either true or false	[29, 39]
Coherence	Biographical integrity	Whether the choice made by surrogates maintains or continues the identity of the patient	[24]
	Coherence with overall life story	Decisions with a high degree of coherence with the rest of a patient’s life story; past statements should be contextualizable in light of the patients life and relationships with others	[7, 24, 25, 30, 38, 39, 46]
	Internally consistent	Past statements should not conflict with one another, or should be consistent with patients lifestyle or current wishes	[12, 13, 22, 27, 28, 30–33, 36, 39, 47]

^1^Many studies however refer to a study by Shalowitz et al. [47], who define surrogate accuracy as “how well surrogates predict patients’ treatment preferences”

### Correspondence standard

In the correspondence view, a good reconstruction process leads to a set of treatment preferences that closely correspond to a patients’ factual treatment preferences. Whether the reconstruction is good is determined by its clarity, convincingness, explicitness, and specificity – with accuracy as the ultimate touchstone epistemic value [22, 25–29, 32, 34]. A casual statement made by a patient to her husband to “not want to live like a vegetable” [25] gives less clear and specific proof of a patient’s reconstructed treatment preferences than statements such as not wanting “dependence on a wheelchair” [41] or not accepting treatment if the result is that the patient “cannot participate in vocational and everyday life activities” [43]. The latter statements are much more specific and thus instructive for treatment.

Proponents of the correspondence approach argue that the accuracy of reconstructed preferences is the epistemic value that best serves the patient’s personal autonomy [26]. The correspondence standard evaluates surrogates and healthcare professionals based on their ability to provide factual proof of a patient’s premorbid wishes. Table 4 shows the attitudes of surrogates and healthcare professionals that define when someone should be considered as an authoritative voice in the reconstructive process when taking up a correspondence approach. Because reconstruction of treatment preferences is mainly based on surrogate testimony, it is essential that surrogates are trustworthy [13, 23, 27, 40, 44]. Accuracy is valued then to protect the patient’s autonomy from overidentification of the surrogate with the patient and a projection of their own preferences on patients [13, 22]. Critical distance is needed to determine the accurate meaning of past statements, which goes along with requirements of honesty and objectivity on the part of the people involved in the reconstruction [22, 25, 27, 37]. Surrogates may be biased or be “misguided” [26] in the sense of deciding based on memories and ideas that do not correspond to the patient’s actual preference, or may have difficulty identifying the differences between their own needs and values and those of the patient [13, 22, 32]. Accuracy is needed so that a surrogate does not seek proof of consent where there actually is none. These leads to correspondence approach to favor parties to a reconstructive process are able to take a position of critical distance. The correspondence view classifies reconstructed treatment preferences according to levels of evidence [25], parallel to the accepted hierarchical framework in which clear and specific ADs have greater epistemic value than a “casual chat” [13, 25, 32, 41]. Some studies set the evidentiary bar as high as needing tangible proof – e.g. a written AD – in order for reconstruction to be valid at all [22, 25]. In addition, many studies argue for a higher probability threshold in certain cases [25, 32]. Notably, the “stronger the evidence that the individual patient would not want to live in an MCS, the more moral authority proxies will have in removing LST” [32]. This is especially the case for younger patients [25], as well as when family members have polarized views regarding appropriate treatment. Rather than choosing a side, the physician should in those cases engage in further communication to “clarify incorrect or unrealistic information” [25].

**Table 4:** Values, norms and criteria used to evaluate families and/or healthcare professionals that engage in a reconstruction of treatment preferences

**View**	**Values**	**Relevance to families and/or healthcare professionals that engage in a reconstruction of treatment preferences**	**References**
Correspondence	Consistency	Consistency across the decisions made by the surrogate	[42]
	Honesty	Not further defined	[28]
	Impartiality	People who do not know the patient are better positioned to make objective decisions	[38]
	Multidisciplinarity	All healthcare disciplines should be involved in reconstruction of treatment preferences	[27, 36, 40]
	Objectivity	Not further defined	[22, 26, 38]
	Other-directed	Taking the patient seriously as another person; surrogates should not project their own treatment preferences on the patient or overidentify with the patient	[22, 41]
	Proximity	Those who are close to the patient are best positioned to interpret the patient’s current wishes; also discussed in light of the risk of projection of surrogates’ wishes on patients	[22, 36]
	Rationality	Surrogates should have the ability to form reflective treatment preferences	[28, 47]
	Sensitivity to emotions	Physicians should be sensitive to strong emotions of surrogates but not equate those with irrationality	[28]
	Trustworthiness	Believe in the good judgment of other co-decision-makers	[13, 24, 28, 41, 45]
Coherence	Constrained creativity, being imaginative	Surrogates are creatively free in the sense that their decisions may reflect, to some extent, the proxy’s own perspective; they are constrained in the sense that they should base themselves on the patient’s life story	[24]
	Familiarity	Those who know the patient best should be involved in reconstruction of treatment preferences; the surrogate must share the lifeworld of the patient, not be a stranger	[24, 41]
Communitarian	Adamancy	Surrogates that come across as assured and reiterate their knowledge of a patient’s treatment preferences	[44]
	Co-constitutive reciprocity	The patient’s family has to be taken seriously as co-constitutive of the patient’s identity; Anyone involved or interested in the patient’s treatment should not treat the patient as if s/he were anonymous; as if the patient is not communally embedded and/or nothing is known about his/her life	[25, 38]
	Respectful of kinship or filial piety	Doing anything to help a family member, not giving up on the patient out of duty	[41, 47]

Adherents of the correspondence view thus adopt language by which treatment preferences can be “attributed” [22, 25] to the patient, “determined” [12, 26–28, 31], or “extrapolated” [22, 25, 27] based on the evidence, rather than reconstructed. All of these terms suggest that there are major contingencies involved in accurately representing the patient. Reconstructed preferences that cannot be tested for correspondence to the patient’s factual treatment preferences are considered a form of “guessing” or “speculating” [13, 22]. Some accounts criticize the entire notion of reconstruction for being "counterfactual" [23, 28, 29, 41], “invented” [22], or “fictionalized” [22]. Because in a process of reconstruction, complete accuracy can never be obtained, it becomes difficult to base any course of treatment on the patient’s wishes on the correspondence view. Nagasawa argues in this vein that the question “what would the patient have wanted, were he awake and competent” itself incites counterfactual thinking based on thought-experiments that can never lead to a reconstruction that accurately corresponds to what the patient would truly have wanted [28]. Those adopting a correspondence standard may run into the particular dilemma of reemergent consciousness, and hence partial decision making capacity, for patients who progress from UWS to MCS. Among authors who appear to adopt the correspondence value of accuracy [13, 29, 32–36], there is an unresolved debate whether priority should be given to past versus current preferences; or alternatively what is called critical versus experiential interests. For patients in UWS, reconstruction amounts to collecting accurate historical information about the patient’s preferences. However, for patients in the MCS, it is argued that current utterances and experiences may also indicate treatment preference [13, 29, 32]. This challenges adherents of the correspondence view to choose which conception of autonomy should be prioritized: precedent autonomy as expressed by the patient’s past wishes, or a patient’s current autonomy as expressed by the patient’s current – however inchoate – indication thereof. Graham argues that surrogate decision-makers should imagine how the patient is experiencing their current life, prioritizing the patient’s experiential interests because these impact their current situation most [13]. Experiential interests entail whatever we currently find exciting, enjoyable, or pleasurable. They contrast with critical interests, which are convictions about what makes a life good on the whole [48]. A dilemma then arises when patients regain intermittent or covert awareness and a conflict arises between the reconstruction based on historical information and their inferred experiential interests based on current observations [13, 31–33, 44]. In patients with covert awareness a response shift may take place, i.e. a “recalibration, reconceptualization or reprioritization of a patient’s values and commitments in response to significant changes in their circumstances, in a way that affects their self-evaluation” [13, 36]. This would entail that the preferences of patients in MCS adapt and evolve, invalidating reconstructed treatment preferences based on who a patient was in the past.

Fins argues that in this case, a mosaic approach should be pursued in which both historical information and present experiences weigh in on the extrapolation of treatment preferences [32]. Scholten et al. argue that current experiences are different from preferences, because preference formation requires the rational ability to understand and reflect upon various treatment options. Because preferences – rather than experiences – better reflect autonomy, and only the past preferences of patients in MCS can be accurately knowable, they argue that priority should be given to past preferences. [34] Hawkins finds this picture to simplistic, and argues that the past should only become relevant to present decision-making when “all or most of […] valued [decision-making] capacities are lost.” [36].

### Coherence standard

The emphasis on accuracy has been criticized by proponents of the coherence view, who argue that a good process of reconstruction consists of looking for consistency between everything that is known about a patient. This view sets out from the empirical claim that people who lapse into DoC are often young and have no clear preferences about what they would want in this situation to begin with. This makes it nonsensical to ask for an accurate correspondence between real preferences and reconstructed preferences, because the former may have never taken shape in the first place. At the same time, by ignoring everything else that is known about the patient, the correspondence approach results in treating most patients as anonymous, decontextualized persons most of the time. Kuczeswki therefore notes that the kind of accuracy and specificity required by the correspondence approach risks brushing aside even the most compelling testimony of surrogates based on having known the patient for years [26]. Coherentists therefore argue that family members and healthcare professionals may validly reconstruct the preferences of patients with DoC based on consistency with the patient’s biography or life narrative [23, 24, 30–32, 37, 38], identity [23, 24, 32], pre-illness personality [38] and lifestyle [22, 27]. It would be morally reasonable to have this biographical information somehow bear on the question of what the patient would have wanted [31]. Coherentism, then, does not claim that accuracy does not matter at all, but rather claims that aiming for a high degree of accuracy is “unlikely to be achieved” [31] and would risk neglecting a patient’s personal identity [24]. Therefore, the question guiding the reconstructive effort should not be how to best (i.e. accurately) respect the patient’s personal autonomy, but what choice sufficiently coheres with the patient’s personal identity [23, 24]. A surrogate may thus reach the conclusion that her brother with PVS would not have wanted to go on like this because it did not fit his “vibrant, clever and passionately spirited” character, coupled with the observation that he “treasured his connection to others”, and his “physical and mental strength” [40].

Accordingly, reconstructed treatment preferences result from telling a consistent story about the patient [23, 24, 31, 32, 38]. This does require creativity and imaginative capacities on the part of surrogates. At the same time creative freedom is restricted based on the biographical information known (Table 4). Proponents of this view admit that out of multiple possible stories, it can be tough to decide which should be considered authoritative [29]. As Blustein points out, “a patient’s general values or traits typically provide proxies with only very indeterminate guidelines for choice in specific circumstances […] In particular, it does not always yield unequivocal answers to questions about appropriate treatment of” DoC patients [23]. There may be “more than one intelligible continuation of [a patient’s life story]” and “that satisfies the condition of narrative fit.” [23] Hence, in the coherentist approach, consistency supersedes accuracy as the criterium by which reconstructed treatment preferences should be justified. Blustein considers this a strength of the coherence approach, because it is unreasonable to expect that surrogates will always be able to come up with the uniquely accurate reconstruction that is required by the correspondence approach. Instead, “decisions that have a high degree of coherence with the rest of a patient’s narrative self-conception are those that are faithful to who the patient was, as expressed in the organizing principles of her life through which she understood herself and her world” [23].The coherence view has been criticized for being untenable in practice. Reconstructing the patient’s preferences based on the patient’s diverse values, beliefs, practices, and prior statements would require an “imaginative effort” that is beyond the capacities of most people [49]. Moreover, the coherence view does not adequately tackle the issue that surrogates may impose their own views on the patient. In this sense, while focusing on accuracy in the reconstructive process guards against biases of the surrogate, reconstructed preferences that are consistent with the patient’s identity may not be as good as accuracy in safeguarding against the surrogate’s biases [12].

### Communitarian standard

The communitarian view disputes the correspondence theorists’ claim that surrogates’ biases are problematic. Communitarianism argues that when reconstructing the treatment preferences of patients with DoC, it is inappropriate to ask surrogates to isolate the patient as an autonomous individual and think about his or her treatment preferences neutrally, i.e., while attempting to set aside their own biases, identities, and interests. Instead, the patients preferences should be understood from within the relations this patient has with the surrogate and other relatives or friends: the preferences of the patient are intertwined with the preferences of the patient’s surrogates. Communitarianism focuses on the continued importance patients have to the people around them, thus doubting the claim that the only things that matters is determining what the patient’s treatment preferences are; it also matters what the people around them think. Kaufman, for instance, writes that for the partner of a patient with DoC remains “a valuable person with a potentially different future, a social being connected by love and personal history to him and to family” [29]. These considerations are not reconcilable with the correspondence view. It also diverges from the coherence standard in the sense that it grants authority to shared identities and communal interests in treatment of the patients [24, 25]. Both the correspondence and coherence views locate the patient’s subjectivity within the patient, which then requires reconstructive efforts when the patient loses the ability to self-express. But in Kaufman’s example above, the subjectivity of patients is “invested in the patient through staff and family action and interpretation” [38]. Incurring a DoC does not take the patient out of the community, does not end the reciprocal relationship, and therefore a DoC is not – as Kaufman argues – “a catalyst that alter[s] the epistemological frame” based upon which moral decisions have to be made [38].

This means that the patient’s reconstructed treatment preferences cannot be obtained separately from the patient’s reciprocal relations with their families or communities [24, 37, 40, 46]. Table 4 shows the attitudes of surrogates and healthcare professionals that define when someone should be considered as an authoritative voice in the reconstructive process from the point of view of communitarianism. Using the communitarian standard, the patient’s preferences are reconstructed based on a sense of commonality and their role within their community. For instance, treatment may be continued for the mother of a child primarily based on the argument that she is still retains her role as someone’s mother. Continuation of treatment may last for as long as this can be justifiable within the reciprocal relation between the patient and the family. Kuczewski notes that when “a surrogate decides after several years of watching an unresponsive patient that it is time to forgo life-sustaining treatment, it is not that the surrogate believes he had previously made a mistake about what the patient would want. Rather the surrogate is imagining an ongoing dialogue between them over the course of those years. And, in that process, they have decided that it makes no sense to continue further aggressive treatment” [25]. Hence, surrogates are seen as “co-constituents” or “continuers” of the patient’s life narrative *in connection*with their own lives [23, 24].Treatment may be continued because the surrogate retains a sense of familial connection to the patient, and out of duty does not give up on the patient [38, 40, 46, 47]. Table 5 schematically shows each of the views present in the literature on reconstruction of treatment preferences in DoC.

**Table 5:** Classifying views on reconstruction

Standard	Moral value	Epistemic value	Temporal focus
Correspondence	Personal autonomy	Accuracy	Past, present or both
Coherence	Integrity of personal identity	Consistency	Critical interests
Communitarian	Continued importance of patient within relational network	Co-constitutive reciprocity	Present

### Empirical material on reconstruction of treatment preferences

The 13 empirical studies were predominantly conducted in the USA, but there was more geographical variation among these studies. Most studies were qualitative (*n*= 9). In addition, we included 1 quantitative study and 3 case reports. The qualitative studies either used interviews [41, 42, 46, 47], or a combination of ethnographic methods [38–40, 45].

The empirical material shows that in practice, past oral statements made by the patient often impact the reconstructive process [8, 41, 43, 45]. However, in practice, such statements are often scrutinized. For instance, if there are ADs, surrogates or physicians occasionally argue these are non-applicable to the situation [8, 41]. If there are no past oral statements by the patient, surrogates are reported to consider the patient’s premorbid identities, life histories and pre-illness personality to justify treatment decisions [38, 41]. In that case, surrogates infer the patient’s dispositions based on knowledge of their likes and desires, such as that a patient “would not have been able to deal with illness or disability” [41].

Additionally, both surrogates and healthcare professionals are found to infer a patient’s preferences based on their current non-verbal behavior and experience of pain [37, 38, 41]. Kuehlmeyer et al. describe that the majority of surrogates extrapolated a will to live based on the patient’s non-verbal behavior alone. [41] When a patient survived complications such as sepsis, surrogates are reported to call the patient a “fighter”, and therefore feel inclined to continue treatment [41]. Conversely, healthcare professionals have been reported to interpret patients’ preferences based on their current responses to pain [37, 38]. Nurses, especially, are described to read treatment preferences off of the behavior of patients with DoC [38].

Surrogates may at some point come to feel or realize that the patient would not have wanted to live in a state of DoC [38, 40]. Family members can “turn a corner”, realizing that it would be better to withdraw or withhold LST at some point [40]. Kitzinger and Kitzinger report that most surrogates initially want LST for their relative, but later come to the position that their relative would not accept living in their current state [8]. Kuehlmeyer et al. report of cases in which this is the other way around: surrogates opt to continue treatment even if the patient has previously indicated these treatment choices to be a red line. [41]

Studies mention treatment decisions being made by a family unit as a whole [8, 39, 42], a patient’s partner [7, 8, 38, 42, 43, 45–47], their parents [7, 8, 39–42, 44–46], their siblings [7, 8, 39, 40, 42, 44, 47], their child and/or multiple children [7, 8, 38, 41, 42, 46], a daughter-in-law [7], or a friend [47]. Often, multiple family members play a role in informal reconstructive processes [8, 39–42], and sometimes, family members disagree amongst each other about what the patient would have wanted [40, 44, 45]. Empirical studies further describe that healthcare professionals sometimes feel that surrogates decide against a patient’s premorbidly expressed wishes [41, 44]. In the eyes of healthcare professionals, surrogates appear to weigh the patient’s expressed will against what they believe is the patient’s recovery potential [7, 8, 38, 39, 41, 42, 44, 46]. Healthcare professionals note that surrogates are often guided by hopeful expectations of recovery, and that this may interfere with an accurate reconstruction of treatment preferences [38]. On the other hand, Kitzinger and Kitzinger report cases in which surrogates felt that healthcare professionals were sometimes keeping patients alive against their wishes, mainly because healthcare professionals feel the “window of opportunity” to legitimately withdraw treatment, e.g. a medical crisis, has closed [8]. Additionally, surrogates are reported to experience guilt towards the patient if they give up on treatment [40, 46, 47]. Lavrijsen et al. describe a case in which treatment of a patient with DoC was continued simply because the family did not want to lose the patient even as the family recognized that the patient experienced a “fate worse than death”, [39] and the physician deemed treatment not in the patient’s interest.

Lastly, several studies show that for many families of patients with DoC the ward and the nurses become their primary social context [44]. Often, nurses get to know the patient and the family more intimately than physicians do [35, 38], and may gain an intimate understanding of the family’s reasons behind certain choices [38].

## Discussion

The thirty studies included in this systematic review mainly show that there are different approaches to address the epistemically difficult task of reconstructing preferences of patients with DoC. Roughly, reconstructed preferences can either be evaluated for their accurate correspondence to the patient’s previously uttered treatment preferences, for their coherence across several sources of biographical information, or guided by the value of the patient’s continued relation with relatives and friends. For patients in UWS, past values are seen as instructive for preference reconstruction, while in accounts on patients in MCS present utterances and experiences are additionally taken into account. When patients reemerge into MCS, this mostly causes a dilemma for the correspondence account. In a sense, reconstruction is the reverse of advance care planning, being retrospective as opposed to forward looking in character.

Healthcare professionals are reported to know of patients’ preferences from surrogates’ testimony or based on direct observation of patients’ behavior. In day-to-day talk, testimony is a ubiquitous and relatively unproblematic source of knowledge. However, when stakes are high – e.g., when considering withdrawal of LST – the epistemic criteria for testimony become more stringent, and accuracy becomes one of the main values in the reconstructive process. Empirical studies on surrogate decision-making have often focused on surrogates’ predictive accuracy [41]. In their influential systematic review, Shalowitz et al. show that next-of-kin surrogates are inaccurate about end-of-life preferences in one third of all cases. [50] Shalowitz et al. have critically asked whether accuracy should indeed be the guiding epistemic criterium for surrogate decision-making. Several studies in this review argue that prioritizing accuracy sets the evidentiary bar so high that – paradoxically – it becomes impossible for an individualized decision to be made. Because patients with DoC are likely to have never formed clear preferences about what they would want in a DoC, [6–8, 24] several studies prefer basing reconstructive efforts on coherence with the patient’s biography or worldview instead of on accuracy [23, 24, 30–32, 37, 38]. However, little is known about the “imaginative effort” [49] by which surrogates may reason from indefinite biographical material to clear-cut treatment preferences. Based on our findings, we classify processes of reconstructing treatment preferences according to the dimensions in Table 5. The empirical material shows that in clinical practice, conflicts may arise if physicians and surrogates approach preference-reconstruction from different viewpoints. For instance, healthcare professionals may feel that surrogates decide against the patient’s premorbid wishes, while surrogates see it as their task to be an advocate of the patient’s interests. Hence, for surrogates, other values may prevail than for physicians or nurses. Studies have indeed found that surrogates conflate surrogacy with advocacy of the patient’s best interests [51]. This supports the basic premise of the correspondence standard that it may be challenging for surrogates to faithfully reconstruct the patient’s wishes [51]. In clinical practice, different approaches may be combined to guide and evaluate reconstructed treatment preferences. We note, however, that inferring treatment goals based on direct observations of patients’ current experiences may be essentially different from obtaining consent [37, 38, 41, 52]. Inferring psychological mental states does not lead to any knowledge of treatment preferences [34, 52]. Epistemological problems remain.

The communitarian approach can be understood as a fundamental critique of the notion that the preferences of patients can somehow be determined in isolation, i.e. apart from their relationship with others. However, there may be at least two different ways of understanding communitarianism, and the distinction between them is not made clear in the literature. First, communitarianism may imply that in addition to the patient’s individual treatment preferences, the preferences of people with whom they are in intimate relationships matter. This line of thought is mostly present in the study of Chen et al., where the holistic wisdom of the family is a factor that is taken into consideration besides the individual’s treatment preferences. [46] Second, communitarianism may be understood as that the individual treatment preferences of the patient are somehow constructed through their relationships. The first view admits that it is not only the patient’s preferences that are important, and this would imply a turn away from prioritizing respect for patient autonomy. The second view of communitarianism – which seems to be Kuczewski’s approach [24] – seems to preserve the narrative that the patient’s autonomous choices do play a role. In Kuczewski’s view, family members imagine themselves to be in an ongoing dialogue with patients, and may ultimately decide to forego treatment based on how they imagine this dialogue to proceed over time.

The notion of response shift is important in relation to this last point. It is likely that prior to acquiring a disability, patients overestimate the degree to which this disability compromises their happiness or well-being [53]. For this reason, Graham has argued that satisfying past preferences of patients with DoC may not be in the patient’s current best interests, because these past preferences are misinformed [13, 54]. Interestingly, a response shift is observed in surrogates of patients with DoC, as they are often reported to continue treatment while at the same time indicating that according to the patient’s past preferences, she would not have wanted to live with the DoC. The surrogates themselves also indicate they would not have wanted to live with a DoC [41, 44]. This suggests that the well-being of patients in a DoC is indeed subject to different considerations than that of healthy adults [52]. However, Kitzinger and Kitzinger reported the reverse process [8].

### Recommendations for clinical practice

Based on the results we distill several important recommendations for healthcare professionals communicating with surrogates of patients with DoC. The way the physician inquires about an incapacitated patient’s treatment preferences is likely to invite the surrogates to respond according to one of the three views which we identified. For instance, asking what the patient *would have wanted* using the past tense may incite the surrogate to reconstruct the patient’s preferences based on past values using a correspondence framework. Asking *who the patient is* will most likely incite the surrogate to respond in a manner befitting the coherence view and provide an account of the patient’s identity. Asking about the patient’s relation with important others may give the physician a sense of what matters to the patient’s community. To obtain a full and rich picture, the physician is advised to ask questions befitting of view, as this allows a triangulation of all perspectives.

Our results point out that surrogates may be too emotional to enact critical distance, which makes preference reconstruction liable to bias [22]. The correspondence standard is meant to correct this by scrutinizing the surrogate’s reconstruction using accuracy as the epistemic touchstone. However, by pursuing accuracy of reconstructed preferences in these circumstances, the physician may come off as wanting to test the surrogate’s trustworthiness or reliability – and consequently coming of as distrustful of the surrogate. With regards to surrogates’ testimony, physicians should therefore neither be “too skeptical nor too gullible” [54]. We advise physicians to not engage in extensive querying of a surrogate’s testimony in a single conversation, and to alternate questions that ‘test’ the surrogate’s accuracy with questions that allow the surrogate to creatively explore what the patient would want or would have wanted. Over time, physicians may gain inductive evidence of the surrogate’s trustworthiness [55]. Reconstructing treatment preferences is, in our view, best approached as a low-pace activity, stretched out over multiple conversations. While preference reconstruction may be seen as the retrospective reverse of advance care planning, analogous to advance care planning, it is wise to engage in periodic review of the reconstructed treatment preferences with surrogates [14].

In practice, we suggest that physicians explicate their own values and rationale for reconstructing treatment preferences, and also ask surrogates to elaborate on their own values and clarify their role. Alternatively, reconstructing the patient’s treatment preferences could also be made the responsibility of a third party on whom the surrogate do not depend for care for their relative with DoC.

### Strengths and limitations

This review identified values and criteria for reconstructing treatment preferences in both conceptual and empirical studies. This helps laying bare normativity in empirical studies while at the same time, identifying practical realities which are overlooked by conceptual work, e.g. the fact that most people do not have specific ADs, that preferences evolve during or as a consequence of DoC, and the tendency of both surrogates and healthcare professionals to focus on directly observable information. However, many of the empirical studies in this review are based on analysis of few cases or single, fairly unique cases. One the one hand, this is a strength, because it captures extreme or deviant cases which are especially illustrative. However, case reports may suffer from publication bias. Often, success stories are published, and these distract from what is commonly dilemmatic in reconstruction of treatment preferences.

Most of the included studies were written within a Western cultural and medical context. As illustrated by one study from China, the communitarian standard may to some extent reflect reconstruction of treatment preferences in non-Western countries, but this review did not identify conceptual studies that can back this claim.

## Conclusion

There is no standard approach to reconstructing treatment preferences. Reconstructive processes may consider past values of patients in DoC, but current experiences gain more weight in the process as patients improve from UWS to MCS. Reconstructed preferences may be evaluated from several epistemic viewpoints. The way in which the physician inquires about an incapacitated patient’s treatment preferences may steer the surrogate’s response.

## Supplementary Information

Supplementary Material 1.

## Data Availability

The datasets used and/or analysed during the current study are available from the corresponding author on reasonable request.
