# Supplementary material for: Reconstruction of patients' treatment preferences in disorders of consciousness: a systematic review
Source: BMC Med Ethics. 2025 Jul 4;26:84. doi: 10.1186/s12910-025-01241-1 (PMC12231754; doi:10.1186/s12910-025-01241-1)
Supplement: Supplementary file 1 — Supplementary Material 1. [file 12910_2025_1241_MOESM1_ESM.docx]

**Supplement 1: search strategy**

| **Database** | **Search Strategy** | **n** |
| --- | --- | --- |
| **PubMed** | (persistent vegetative state[Mesh] OR (vegetative[tiab] AND (state*[tiab] OR status[tiab] OR condition[tiab])) OR (unresponsive[tiab] AND wakefulness[tiab]) OR (coma[tiab] AND vigil[tiab]) OR apallic[tiab] OR (akinetic[tiab] AND mutism[tiab]) OR akinetic mutism[Mesh] OR consciousness disorders[Mesh] OR disorder of consciousness[tiab] OR disorders of consciousness[tiab] OR consciousness disorder*[tiab] OR minimal conscious*[tiab] OR minimally conscious[tiab] OR irreversibly coma*[tiab] OR irreversible coma*[tiab])  AND  (Decision making[Mesh] OR "Personal Autonomy"[Mesh] OR decision making[tiab] OR substitute judgment*[tiab] OR substituted judgement*[tiab] OR substituted judgment*[tiab] OR autonomy[tiab] OR best interest*[tiab] OR surrogate*[tiab] OR family[tiab] OR families[tiab] OR family[Mesh] OR Proxy[tiab] OR third-party consent[mesh] OR third party consent[tiab] OR “Advance Care Planning”[mesh] OR “Advance Care Planning”[tiab] OR “Care Planning”[tiab]) | 4.738 |
| **Embase** | (persistent vegetative state/ OR minimally conscious state/ OR (vegetative.ti,ab,kf. AND (state*.ti,ab,kf. OR status.ti,ab,kf. OR condition.ti,ab,kf.)) OR (unresponsive.ti,ab,kf. AND wakefulness.ti,ab,kf.) OR (coma.ti,ab,kf. AND vigil.ti,ab,kf.) OR apallic.ti,ab,kf. OR (akinetic.ti,ab,kf. AND mutism.ti,ab,kf.) OR akinetic mutism/ OR consciousness disorders/ OR disorder of consciousness.ti,ab,kf. OR disorders of consciousness.ti,ab,kf. OR consciousness disorder*.ti,ab,kf. OR minimal conscious*.ti,ab,kf. OR minimally conscious.ti,ab,kf. or irreversibly coma*.ti,ab,kf. OR irreversible coma*.ti,ab,kf.)  AND  (decision making/ or clinical decision making/ or ethical decision making/ or family decision making/ or medical decision making/ or patient decision making/ or shared decision making/ OR  personal autonomy/ OR patient right/ or patient autonomy/ OR decision making.ti,ab,kf. OR substitute judgment*.ti,ab,kf. OR substituted judgement*.ti,ab,kf. OR substituted judgment*.ti,ab,kf. OR autonomy.ti,ab,kf. OR best interest*.ti,ab,kf. OR surrogate*.ti,ab,kf. OR family.ti,ab,kf. OR families.ti,ab,kf. OR family/ OR Proxy.ti,ab,kf. OR informed consent/ OR third party consent.ti,ab,kf. OR advance care planning.ti,ab,kf. OR advance directive.ti,ab,kf. OR advance directive adherence.ti,ab,kf.)  **Literature with a Conference abstract status was omitted from the search.** | 2.790 |
| **Cochrane** | ([mh "persistent vegetative state"] OR (vegetative:ti,ab,kw AND (state*:ti,ab,kw OR status:ti,ab,kw OR condition:ti,ab,kw)) OR (unresponsive:ti,ab,kw AND wakefulness:ti,ab,kw) OR (coma:ti,ab,kw AND vigil:ti,ab,kw) OR apallic:ti,ab,kw OR (akinetic:ti,ab,kw AND mutism:ti,ab,kw) OR [mh "akinetic mutism"] OR [mh "consciousness disorders"] OR "disorder of consciousness":ti,ab,kw OR "disorders of consciousness":ti,ab,kw OR ("consciousness" NEXT disorder*):ti,ab,kw OR ("minimal" NEXT conscious*):ti,ab,kw OR "minimally conscious":ti,ab,kw OR ("irreversibly" NEXT coma*):ti,ab,kw OR ("irreversible" NEXT coma*):ti,ab,kw)  AND  ([mh "Decision making"] OR "decision making":ti,ab,kw OR ("substitute" NEXT judgment*):ti,ab,kw OR ("substituted" NEXT judgement*):ti,ab,kw OR ("substituted" NEXT judgment*):ti,ab,kw OR autonomy:ti,ab,kw OR ("best" NEXT interest*):ti,ab,kw OR surrogate*:ti,ab,kw OR family:ti,ab,kw OR families:ti,ab,kw OR [mh family] OR Proxy:ti,ab,kw OR [mh "third-party consent"] OR "third party consent":ti,ab,kw OR [mh “Advance Care Planning”] OR “Advance Care Planning”:ti,ab,kw) | 120 |
| **Web of Science** | TS=(((vegetative AND (state* OR status OR condition)) OR (unresponsive AND wakefulness) OR (coma AND vigil) OR apallic OR (akinetic NEAR/3 mutism) OR “consciousness disorders” OR “disorder of consciousness” OR “disorders of consciousness” OR “consciousness disorder*” OR “minimal conscious*” OR “minimally conscious” OR “irreversibly coma*” OR “irreversible coma*”)  AND  (“Decision making” OR "Personal Autonomy" OR “substitute judgment*” OR “substituted judgement*” OR “substituted judgment*” OR autonomy OR “best interest*” OR surrogate* OR family OR families OR family OR Proxy OR “third-party consent” OR “Advance Care Planning”)) | 2.005 |
| **CINAHL** | (((MH "Persistent Vegetative State") OR (MH "Minimally Conscious State") OR (MH "Coma") OR (MH "Consciousness Disorders") OR (MH "Unconsciousness+") OR TI((vegetative AND (state* OR status OR condition)) OR (unresponsive AND wakefulness) OR (coma AND vigil) OR apallic OR (akinetic AND mutism) OR disorder of consciousness OR disorders of consciousness OR consciousness disorder* OR minimal conscious* OR minimally conscious OR irreversibly coma* OR irreversible coma*)) OR AB((vegetative AND (state* OR status OR condition)) OR (unresponsive AND wakefulness) OR (coma AND vigil) OR apallic OR (akinetic AND mutism) OR disorder of consciousness OR disorders of consciousness OR consciousness disorder* OR minimal conscious* OR minimally conscious OR irreversibly coma* OR irreversible coma*)))  AND  (((MH "Decision Making, Family") OR (MH "Decision Making, Patient+") OR (MH "Decision Making, Ethical") OR (MH "Decision Making, Shared") OR (MH "Decision Making, Clinical+") OR (MH "Decision Making") OR (MH "Dissent and Disputes+") OR (MH "Uncertainty") OR (MH "Patient Autonomy") OR (MH "Relational Autonomy") OR (MH "Autonomy") OR (MH "Patient-Family Relations") OR (MH "Family") OR (MH "Consent") OR (MH “Advance Care Planning”) OR TI (decision making OR substitute judgment* OR substituted judgement* OR substituted judgment* OR autonomy OR best interest* OR surrogate* OR family OR families OR Proxy OR third party consent OR Advance Care Planning) OR AB(decision making OR substitute judgment* OR substituted judgement* OR substituted judgment* OR autonomy OR best interest* OR surrogate* OR family OR families OR Proxy OR third party consent OR Advance Care Planning))) | 1.184 |
| **PsycINFO** | (consciousness disorders/ or coma/ or unconsciousness/ OR consciousness states/ OR (vegetative.ti,ab,id. AND (state*.ti,ab,id. OR status.ti,ab,id. OR condition.ti,ab,id.)) OR (unresponsive.ti,ab,id. AND wakefulness.ti,ab,id.) OR (coma.ti,ab,id. AND vigil.ti,ab,id.) OR apallic.ti,ab,id. OR (akinetic.ti,ab,id. AND mutism.ti,ab,id.) OR disorder of consciousness.ti,ab,id. OR disorders of consciousness.ti,ab,id. OR consciousness disorder*.ti,ab,id. OR minimal conscious*.ti,ab,id. OR minimally conscious.ti,ab,id. OR irreversibly coma*.ti,ab,id. OR irreversible coma*.ti,ab,id.)  AND  (decision making/ or exp choice behavior/ or ethical decision making/ OR family/ OR informed consent/ OR decision making.ti,ab,id. OR substitute judgment*.ti,ab,id. OR substituted judgement*.ti,ab,id. OR substituted judgment*.ti,ab,id. OR autonomy.ti,ab,id. OR best interest*.ti,ab,id. OR surrogate*.ti,ab,id. OR family.ti,ab,id. OR families.ti,ab,id. OR Proxy.ti,ab,id. OR third party consent.ti,ab,id. OR advance care planning.ti,ab,id. OR advance directive.ti,ab,id. OR advance directive adherence.ti,ab,id.) | 1.051 |
| **Sociological abstracts** | (MAINSUBJECT.EXACT("Unconsciousness")  OR  abstract((vegetative AND (state* OR status OR condition)) OR (unresponsive AND wakefulness) OR (coma AND vigil) OR apallic OR (akinetic AND mutism) OR disorder of consciousness OR disorders of consciousness OR consciousness disorder* OR minimal conscious* OR minimally conscious OR irreversibly coma* OR irreversible coma*) OR title((vegetative AND (state* OR status OR condition)) OR (unresponsive AND wakefulness) OR (coma AND vigil) OR apallic OR (akinetic AND mutism) OR disorder of consciousness OR disorders of consciousness OR consciousness disorder* OR minimal conscious* OR minimally conscious OR irreversibly coma* OR irreversible coma*) OR summary((vegetative AND (state* OR status OR condition)) OR (unresponsive AND wakefulness) OR (coma AND vigil) OR apallic OR (akinetic AND mutism) OR disorder of consciousness OR disorders of consciousness OR consciousness disorder* OR minimal conscious* OR minimally conscious OR irreversibly coma* OR irreversible coma*) OR title(end of life))  AND  (MAINSUBJECT.EXACT("Will") OR MAINSUBJECT.EXACT("Autonomy") OR MAINSUBJECT.EXACT("Participative decision making") OR MAINSUBJECT.EXACT("Decision making") OR MAINSUBJECT.EXACT("Medical decision making") OR MAINSUBJECT.EXACT("Families & family life") OR MAINSUBJECT.EXACT("Informed consent") OR  abstract("decision making" OR "substitute judgment*" OR "substituted judgement*" OR "substituted judgment*" OR autonomy OR "best interest*" OR surrogate* OR family OR families OR Proxy OR "third party consent" OR “Advance Care Planning”) OR title("decision making" OR "substitute judgment*" OR "substituted judgement*" OR "substituted judgment*" OR autonomy OR "best interest*" OR surrogate* OR family OR families OR Proxy OR "third party consent" OR “Advance Care Planning”) OR summary("decision making" OR "substitute judgment*" OR "substituted judgement*" OR "substituted judgment*" OR autonomy OR "best interest*" OR surrogate* OR family OR families OR Proxy OR "third party consent" OR “Advance Care Planning”)) | 531 |

**Supplement 2: eligibility criteria**

A study was included if it met the following criteria:

- the study concerns patients with DoC, including coma, the unresponsive wakefulness syndrome (UWS), and the minimally conscious state (MCS);
- the study concerns adult patients of 18 years old or older;
- the study either empirically, normatively or conceptually describes or assesses reconstruction of treatment preferences;
- for empirical studies no restrictions were applied regarding the study design;
- the study describes or assesses the role of family members with regard to surrogate treatment decision-making;
- the study either concerns the acute, post-acute or chronic care phase;
- the study is written in English.

A study was excluded based on the following exclusion criteria:

- Editorials, newspaper articles and abstracts are excluded.
- The study is published prior to 1 April 1972, when the term Persistent Vegetative State was introduced.
- the study concerns patients that are brain death;
- the study is about decision-making in transplantation medicine;
- the study concerns pediatric patients;
- the study solely focuses on psychological or emotional distress and needs of healthcare professionals or surrogate decision makers;
- the study focuses on the contents and/or application of the law or it solely describes and/or assesses a legal case or decision.

**Supplement 3. Methodological quality assessment of included studies using Mixed Methods Appraisal Tool (MMAT) 2018 and JBI**

| **Study** | **SQ^a^** | | **Qualitative^b^** | | | | | **Quantitative descriptive^c^** | | | | | **JBI Case report^d^** | | | | | | | | **Quality^e^** |
| --- | --- | --- | --- | --- | --- | --- | --- | --- | --- | --- | --- | --- | --- | --- | --- | --- | --- | --- | --- | --- | --- |
|  | S1 | S2 | 1.1 | 1.2 | 1.3 | 1.4 | 1.5 | 4.1 | 4.2 | 4.3 | 4.4 | 4.5 | 1 | 2 | 3 | 4 | 5 | 6 | 7 | 8 |  |
| **Chen et al. (2023)** | Y | Y | Y | Y | C | N | Y |  |  |  |  |  |  |  |  |  |  |  |  |  | W |
| **Kaufman (2000)** | Y | Y | Y | Y | C | N | Y |  |  |  |  |  |  |  |  |  |  |  |  |  | W |
| **Kitzinger et al. (2013)** | Y | Y | N | N | Y | Y | N |  |  |  |  |  |  |  |  |  |  |  |  |  | W |
| **Kitzinger et al. (2016)** | Y | Y | Y | Y | Y | Y | Y |  |  |  |  |  |  |  |  |  |  |  |  |  | S |
| **Kuehlmeyer et al. (2012)** | Y | Y | Y | Y | Y | Y | Y |  |  |  |  |  |  |  |  |  |  |  |  |  | S |
| **Lavrijsen et al (2005)** | C | N | N | N | N | N | N |  |  |  |  |  |  |  |  |  |  |  |  |  | W |
| **Malhi et al. (2023)** | Y | Y | Y | N | N | N | Y |  |  |  |  |  |  |  |  |  |  |  |  |  | W |
| **Picozzi et al. (2021)** | Y | N | Y | N | Y | Y | Y |  |  |  |  |  |  |  |  |  |  |  |  |  | M |
| **Span-Sluyter et al. (2018)** | Y | N | Y | N | Y | C | C |  |  |  |  |  |  |  |  |  |  |  |  |  | W |
| **Jox et al. (2015)** | Y | Y |  |  |  |  |  | Y | C | N | Y | Y |  |  |  |  |  |  |  |  | M |
| **Baek (2016)** |  |  |  |  |  |  |  |  |  |  |  |  | Y | C | Y | Y | Y | Y | Y | N | M |
| **Crow (2006)** |  |  |  |  |  |  |  |  |  |  |  |  | N | C | Y | N | N | N | Y | Y | W |
| **Hunter (1985)** |  |  |  |  |  |  |  |  |  |  |  |  | Y | Y | Y | Y | N | N | C | N | W |

Y yes; N no; C can’t tell; n/a not applicable

*: Other methods would have been more suitable to answer the research question

#: self-selection with risk of bias

+: The methodology is only suitable for a part of the research question

**a. Screening questions (SQ)**

S1: Are there clear research questions?

S2: Do the collected data allow to address the research questions?

**b. Qualitative**

Q1.1: Is the qualitative approach appropriate to answer the research question?

Q1.2: Are the qualitative data collection methods adequate to address the research question?

Q1.3: Are the findings adequately derived from the data?

Q1.4: Is the interpretation of results sufficiently substantiated by data?

Q1.5: Is there coherence between qualitative data sources, collection, analysis and interpretation?

**c. Quantitative descriptive**

Q4.1: Is the sampling strategy relevant to address the research question?

Q4.2: Is the sample representative of the target population?

Q4.3: Are the measurements appropriate?

Q4.4: Is the risk of nonresponse bias low?

Q4.5: Is the statistical analysis appropriate to answer the research question?

**d. JBI Case report**

Q1: Where the patient’s demographic characteristics clearly described?

Q2: Was the patient’s history clearly described and presented as a timeline?

Q3: Was the current clinical condition of the patient on presentation clearly described?

Q4: Were diagnostic tests or assessment methods and the results clearly described?

Q5: Was the intervention(s) or treatment procedure(s) clearly described?

Q6: Was the post-intervention clinical condition clearly described?

Q7: Were adverse events (harms) or unanticipated events identified and described?

Q8: Does the case report provide takeaway lessons?

**e. Quality**

S: Strong

W: Weak

M: Moderate
